# Necrotizing Orchitis due to COVİD-19

**DOI:** 10.1590/0037-8682-0408-2021

**Published:** 2021-09-06

**Authors:** Serdar Aslan, Uluhan Eryuruk

**Affiliations:** 1 Giresun University, Faculty of Medicine, Department of Radiology, Giresun, Turkey.

**FIGURE 1:**
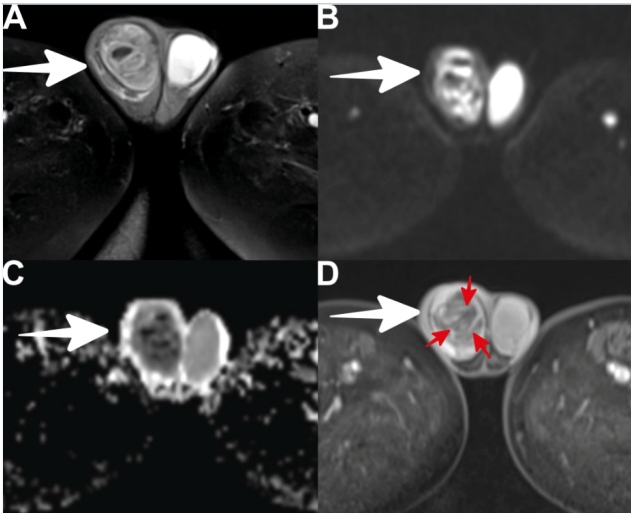
**A -** Axial T2-weighted images showing an increased size of the right testis, heterogeneous changes in parenchymal signals, and cystic areas (arrow). **B and C -** Diffusion-weighted images and apparent diffusion coefficient (ADC) maps showing diffusion restriction of the right testis (arrow). **D -** Axial contrast-enhanced images showing the loss of contrast enhancement (arrow) and areas of septal enhancement (red arrows) in the right testis.

A 33-year-old man who had been hospitalized for ten days due to COVID-19 was referred to us with complaints of swelling, pain, and temperature increase in the right scrotum for the last two days. Physical examination revealed an erythematous appearance, swelling, and pain in the right scrotum, suggestive of acute scrotum. Ultrasonography (US) revealed an increase in the size of the right testis, a heterogeneous appearance of the testicular parenchyma, loss of blood supply in focal places, and dense cystic areas, suggestive of necrosis. Scrotal magnetic resonance imaging (MRI) was performed because US could not differentiate between a testicular mass, torsion, and orchitis. On scrotal MRI, an increase in the size of the right testis, heterogeneous signal and cystic areas in the testicular parenchyma, diffusion restriction, and septal enhancement with loss of enhancement were observed (Figure 1A-D). Necrotizing orchitis was considered in the foreground, and the diagnosis was confirmed as a result of the histopathological evaluation.

It is known that SARS-CoV-2, the causative agent of COVID-19, infects host cells by binding to angiotensin-converting enzyme-2 (ACE-2) receptors^1^. It has been reported that the ACE-2 receptor is expressed in Leydig cells in the testicles; therefore, the virus may cause damage by affecting the testicles^2^. Although orchiepididymitis cases due to COVID-19 have been described in the literature before, to the best of our knowledge, ours is the first necrotizing orchitis case due to COVID-19. Clinicians and radiologists should be aware of necrotizing orchitis, which, although rare, may develop in COVID-19 cases.
